# Automated Antithrombin Activity Detection with Whole Capillary Blood Based on Digital Microfluidic Platform

**DOI:** 10.3390/mi16070785

**Published:** 2025-06-30

**Authors:** Dongshuo Li, Hanqi Hu, Hanzhi Zhang, Lei Shang, Tao Zhao, Qingchen Zhao, Shuhao Zhang, Fucun Ma, Guowei Liang, Rongxin Fu, Xuekai Liu

**Affiliations:** 1 Department of Clinical Laboratory, Aerospace Center Hospital, Beijing 100049, China; lidongshuo1015@163.com (D.L.); leishamgsmile@foxmail.com (L.S.); mafucun721@126.com (F.M.); 2 School of Medical Technology, Beijing Institute of Technology, Beijing 100081, China; 15258319540@163.com (H.H.); zhanghz20020608@163.com (H.Z.); 3220242885@bit.edu.cn (T.Z.); 13231821195@163.com (Q.Z.); zhangruanshi@163.com (S.Z.)

**Keywords:** AT, point-of-care testing, microfluidics, in vitro diagnostics, optical biosensors

## Abstract

Antithrombin (AT) plays a crucial role in the human anticoagulant system and has extensive clinical applications. However, traditional detection methods often require large sample volumes, complex procedures, and lengthy processing times. Methods: We integrated digital microfluidics technology with AT detection to develop a point-of-care testing (POCT) device that is user-friendly and fully automated for real-time AT testing. Results: This device allows for automation and enhanced adaptability to various settings, requiring only a minimal sample volume (whole capillary blood), thereby omitting steps such as plasma separation to save time and improve clinical testing efficiency. Comparisons with conventional AT activity detection methods demonstrate a high degree of consistency in the results obtained with this device. Conclusion: The AT detection system we developed exhibits significant effectiveness and holds substantial research potential, positioning it to evolve into a clinically impactful POCT solution for AT assessment.

## 1. Introduction

AT is a serine protease inhibitor synthesized by the liver and endothelial cells, accounting for approximately 70% of the overall anticoagulant activity [1,2,3]. Heparin, a commonly used anticoagulant in clinical practice, significantly enhances the activity of AT by over a thousandfold upon binding [4,5]. A decrease in AT activity consequently diminishes the clinical efficacy of heparin [6,7]. The coagulation and anticoagulation systems in the body maintain a dynamic equilibrium; thus, a decline in AT levels promotes thrombus formation and increases the risk of venous thromboembolism (VTE), particularly in pregnant women who are inherently in a hypercoagulable state [8,9]. This decline notably elevates the incidence of thrombotic complications during pregnancy [10,11,12]. Additionally, AT levels can serve as a useful indicator of the severity of liver injury and can assist in the categorization of renal diseases [13,14,15]. Therefore, the assessment of AT levels is crucial for monitoring the efficacy of heparin therapy, screening high-risk populations, evaluating thrombosis risk, guiding anticoagulant treatment and managing complications, aiding in the diagnosis of liver and kidney diseases, and preventing disease progression [16,17,18].

**Figure 1:**
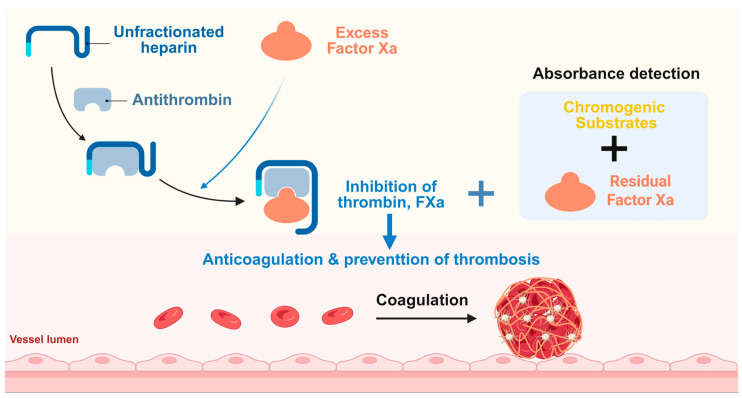
Schematic diagram of the AT detection method.

Currently, the clinical detection of AT includes both antigen assays and activity assays [19]. Active assays are utilized for the quantitative assessment of AT activity in inhibiting coagulation factors in the blood. Clinically, luminescent substrate methods are commonly employed for AT activity quantification. This approach involves adding an excess of thrombin to the tested plasma, leading to the formation of a 1:1 complex between the AT and thrombin. The remaining thrombin subsequently hydrolyzes the luminescent substrate S-2238, generating yellow p-nitroaniline (pNA). The extent of the color change correlates with enzyme activity, allowing for the evaluation of enzyme performance through the measurement of the absorbance change in the yellow color, as illustrated in Figure 1. Ultimately, the activity is determined by comparing the absorbance change following the addition of the luminescent substrate reagent with calibrated plasma. Despite the widespread application of the luminescent substrate method in clinical diagnostics, several notable limitations exist. Firstly, this method relies heavily on the collection of plasma samples; any errors during the preparation, handling, or transport of blood specimens can adversely affect the quality of the samples, thus impacting the test results. The implementation of POCT could help reduce sample transport and management costs, as well as decreasing waiting times for test results. Secondly, current coagulation sampling typically involves the use of citrate anticoagulants, requiring nearly 3 mL of blood. Minimizing the volume of blood needed for testing would significantly enhance clinical management efficiency and would be particularly beneficial for patients for whom blood collection is difficult. Furthermore, AT detection primarily targets plasma samples, necessitating the centrifugation of blood to separate plasma and cellular components. If whole blood could effectively be used to detect AT activity, this would considerably reduce the time costs associated with centrifugation and other processing steps. Additionally, conventional clinical testing methods often rely on large equipment, which requires a high level of expertise from operators, thus constraining their applicability in high-throughput and rapid testing scenarios.

With the rising incidence of thrombotic diseases, there is an increasing clinical demand for the detection of AT activity. Due to the numerous limitations associated with conventional testing methods, there is an urgent need to develop a more efficient, precise, and convenient approach for AT detection. POCT aims to facilitate rapid and convenient clinical evaluations, and it has been widely implemented in areas such as clinical monitoring, inspection and quarantine, and home healthcare [20,21,22]. Digital microfluidics (DMF) technology, characterized by its miniaturization, integration, and automation capabilities, aligns well with the evolving demands of POCT and plays a significant role in optimizing clinical diagnostics [23,24,25]. DMF represents a novel droplet manipulation technology that allows for the independent control of discrete droplets, typically in the microliter to picolitre range, thereby requiring minimal sample volumes [26,27]. Through dielectric wetting, DMF can alter the wettability of droplets on a hydrophobic surface, enabling precise control over their distribution, movement, merging, and splitting, in an integrated and automated manner [28,29,30]. The micro-miniaturized, automated, and high-throughput attributes of DMF technology mimic in vivo conditions, providing a new platform for AT activity detection [31,32]. Compared to traditional methods, digital microfluidic systems offer simplified operations, reduced sample and reagent consumption, accelerated testing speeds, independence from large, sophisticated equipment, and enhanced adaptability across diverse scenarios [33]. These advantages render DMF technology particularly suitable for the prompt detection of AT activity, significantly improving the efficiency and adaptability of these tests [34,35].

In this study, we develop a digital microfluidic-based system for the detection of AT activity, enabling the automated, accurate, and rapid assessment of AT activity levels. The system allows for the pre-programmed movement and mixing of droplets, facilitating parallel and multiplex detection without manual intervention. First, we validated the operability and accuracy of our system using plasma samples. Subsequently, we found that the appropriate dilution of venous whole blood could mitigate interference from red blood cells, a method equally applicable to capillary blood. This enables direct detection of AT activity in capillary whole blood without centrifugation, enhancing both the portability of the system and patient acceptability, thereby better meeting the needs of at-home testing. The system greatly shortens the detection time for AT, which is more in line with the requirements of POCT detection. Throughout the reaction process, we employed absorbance detection equipment to monitor changes in absorbance in real time, thereby quantitatively analyzing the correlation between AT activity levels and the changes in absorbance over time. To validate the consistency of our detection method with conventional techniques, we conducted a correlation analysis between our results and those obtained via conventional methods, which demonstrated the strong clinical relevance of our approach. This research not only contributes to the advancement of clinical diagnostic technologies but also provides crucial insights for the promotion and development of digital microfluidic systems in biomedical applications.

## 2. Materials and Methods

### 2.1. Reagents and Sample Collection

The factor diluent (Product SKU 0009757600), Xa factor reagent (Product SKU 0020300400), and colorimetric substrate (Product SKU 0020020100) were all procured from Werfen Medical (Instrumentation Laboratory Company, 180 Hartwell Road, Bedford, MA, USA). All subjects provided approximately 3 mL of fasting venous blood from the antecubital vein (blood-to-anticoagulant ratio = 9:1). The collection tube was then placed in a centrifuge at room temperature and centrifuged at 3000 rpm for 15 min. Following plasma separation, a portion of the supernatant was collected for subsequent detection using the microfluidic system, while the remaining sample was subjected to analysis using a fully automated coagulation analyzer (ACL TOP750, Instrumentation Laboratory Company, 180 Hartwell Road, Bedford, MA, USA) to measure AT activity levels, with a normal reference range of 80–120%. After detection, the upper plasma and the lower whole blood cells were inverted and mixed again to obtain a venous whole blood sample, and a certain volume was withdrawn and preserved in an EP tube (Axygen MCT-150-C, Silicon Valley, MA, USA) for subsequent detection of AT activity in venous whole blood by the microfluidic system. To validate the equivalence of capillary blood and venous blood in AT activity detection, we enrolled an additional cohort of participants and collected 20 μL of peripheral blood from the fingertip of all subjects following venipuncture at the cubital fossa. Since the collected capillary blood does not contain any anticoagulant components, it needs to be added to the microfluidic system for detection immediately after collection.

### 2.2. Fabrication of Microfluidic Chips

The experiment utilized indium tin oxide (ITO) glass, known for its excellent optical transparency, as the substrate for the microfluidic chip. The substrate was prepared using magnetron sputtering technology. Subsequently, the electrode array on the chip was fabricated through photolithography and etching processes. A dielectric layer of Parylene-C material was then deposited on the surface of the electrode array via a coating deposition method. Finally, a hydrophobic layer was created on the chip by spinning a uniform coat of 1% Teflon-AF 1600 solution, which was subsequently dried and cooled to produce the experimental chip. The detailed synthesis protocol followed methods described in references [36,37,38].

### 2.3. Optical Absorbance Detection Principle and Procedure

**Figure 2:**
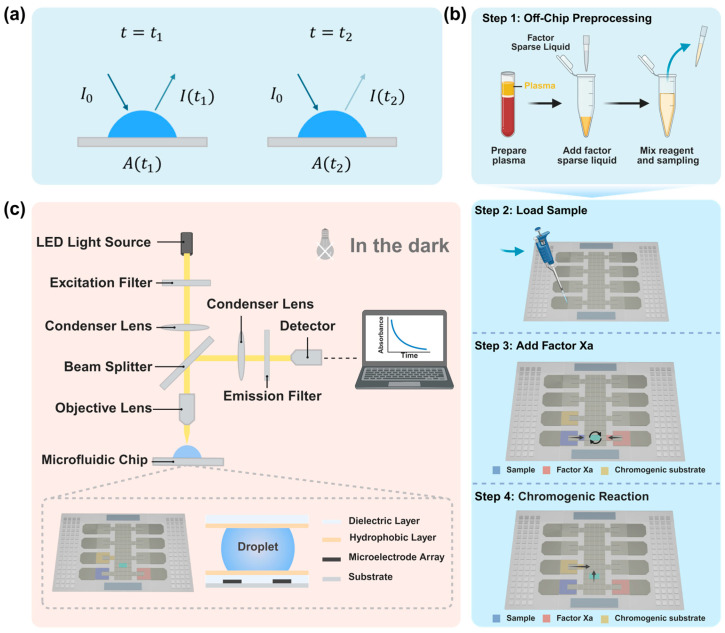
Schematic diagrams of the system’s components and detection principles are presented. (**a**) Schematic representation of the principle of absorbance change detection. (**b**) Illustration of the detection process on the chip. Step 1: following the separation of plasma outside the chip, a factor diluent is introduced as the sample for analysis. Step 2: the prepared sample is applied to the surface of the chip. Step 3: the sample is mixed with Factor Xa to activate the components. Step 4: the resultant mixture is reacted with a chromogenic substrate, and absorbance changes are monitored using an absorbance detection system. (**c**) Schematic diagram of the absorbance detection system’s structure.

The detection of changes in the absorbance of droplets on a chip is illustrated in Figure 2a. In our study, Beer’s Law was applied to quantitatively assess optical absorption following the biochemical reaction. As a foundational principle in absorption spectrophotometry, colorimetric analysis, and photoelectric colorimetry, Beer’s Law is widely used for optical quantification across a range of media, including gases, liquids, solids, molecules, atoms, and ions.

Although conventionally employed in transmission-based measurements of transparent samples, we adapted Beer’s Law to a hybrid optical configuration tailored to our system. Specifically, a normally incident beam passes through the liquid layer, is reflected by the bottom electrode of the chip, and then traverses the liquid a second time before being captured by the optical detection system. Ignoring minor absorption contributions from the chip’s top glass layer, this design effectively constitutes a combined reflection–transmission pathway.

During the initial transmission, light absorption follows classical Beer–Lambert behavior, with absorbance proportional to both analyte concentration and optical path length. Upon reflection, the beam undergoes a second pass through the liquid, introducing an additional absorbance component and incorporating the reflectivity of the chip’s base electrode. To account for this, we reformulate the modified Beer’s Law as follows:

(1) $$I_{OUT}=I_{IN}e^{-\alpha l}r$$

where $I_{IN}$ is the incident light intensity, $I_{OUT}$ is the measured reflected intensity, α denotes the absorption coefficient of the solution, l is the total pass path length, and r is the reflectance of the electrode. Since  r is a constant, the linear correlation between $I_{OUT}$ and $e^{-\alpha l}$ is preserved, validating the continued use of Beer’s Law under this optical configuration. According to Beer’s Law, the real-time absorbance, *A*(*t*), is calculated using the following equation:

(2) $$A(t)=-\mathrm{lg}\left(\frac{I(t)}{I_{0}}\right)$$

In this context, $I_{0}$ represents the intensity of the incident light. I(t) denotes the intensity of incident light at time *t*, which is partially absorbed by pNA molecules. Assuming that the number of photons passing through unit area *A* per unit time is $N_{photons}$, the total optical energy *P* can be expressed as follows:

(3) $${P=N}_{photons}\times h\times f$$

The term *h* represents the Planck constant, and *f* represents the frequency of light, as defined by the intensity of light, I=P/A. The relationship between light intensity and the number of photons passing through the unit area per unit time can be expressed as follows:

(4) $$N_{photons}=\frac{I\times A}{h\times f}$$

Thus, the rate of change in absorbance is obtained with $t_{0}$ = 0 s as the baseline for absorbance:

(5) $$△A=A(t_{1})-A(t_{0})=\mathrm{lg}\left(\frac{t_{0}}{t_{1}}\right)=\mathrm{lg}\left(\frac{N_{photons}(t_{0})}{N_{photons}(t_{1})}\right)$$

Therefore, it is sufficient to examine the number of transmitted photons at $t_{0}$ = 0 s and $t_{1}$ = 60 s to determine the change in absorbance.

**Figure 3:**
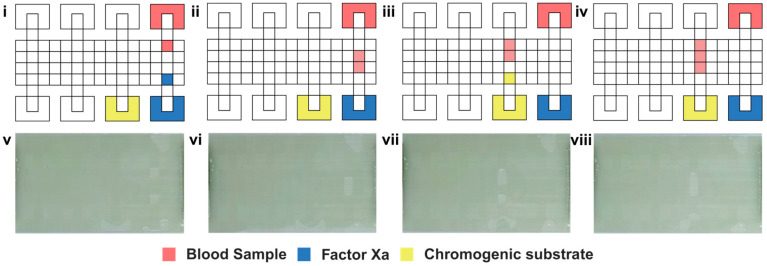
Schematic and actual images of the chip’s reaction when plasma is used as the test sample: (i and v) droplet generation, (ii and vi) reacting with Factor Xa, (iii and vii) moving to the next reaction area, and (iv and viii) mixing with chromogenic substrate.

The experiment was conducted on a digital microfluidic chip based on ITO glass, with absorbance detection equipment employed to record real-time variations in absorbance intensity within the reaction region. As illustrated in Figure 2b, the initial step involved preprocessing the sample externally: 8 μL of the supernatant obtained from centrifuged plasma was mixed with 320 μL of factor dilution buffer. After homogenization, a specified volume of the resulting mixture was extracted. Subsequently, the mixed solution, Xa factor reactant, and chromogenic substrate were each placed in distinct reservoir electrodes. The hardware was powered through a software interface to facilitate the separation of experimental droplets in a volume ratio of 1:1:1, drawing the liquid to the midpoint of the reservoir electrodes located between the upper and lower plates. The schematic diagram and the physical representation are illustrated in Figure 3. Following dilution of whole capillary blood samples at varying ratios and subsequent mixing with the factor-specific diluent, the remaining processing steps were consistent with the plasma protocol.

Before initiating the reagent mixing reaction, the digital microfluidic device was positioned beneath the absorbance detection system. The focal length of the detection lens was adjusted to align with the experimental droplets, ensuring the linear detection of both incident and transmitted light. The light source voltage was calibrated so that the observed aperture precisely covered the reaction droplet following the reaction. To mitigate the effects of external illumination on absorbance measurements, the entire system was operated within a darkroom environment.

### 2.4. Optical Detection System

To enable real-time monitoring of absorbance during the reaction process, we developed a high-precision absorbance detection system, as illustrated in Figure 2c. This module comprises a full-spectrum white-light LED source (400–730 nm, Zhuhai Huayihan, Zhuhai, China), a 405 nm bandpass filter, a dichroic mirror, a photon detector (H11890-01, Hamamatsu, Japan), and a heat sink. The system is powered by a regulated DC power supply (UTP3300TFL-II, UNI-T, Dongguan, China) to ensure the stability of the light source, and active temperature control (±0.5 °C) is achieved by installing the heat sink above the LED light source, effectively mitigating detection errors caused by thermal drift. The incident light path begins with emission from the white-light LED, which is filtered through the 405 nm bandpass filter to select monochromatic excitation light (center wavelength: 405 nm; half-peak width: 10 nm). This light is then focused onto the microfluidic chip reaction chamber via the dichroic mirror. The colorimetric reaction generates pNA, which exhibits characteristic absorbance under 405 nm excitation. The reflected light signal is directed by the dichroic mirror to the photon detector. Within the photon counter, the photomultiplier tube (PMT) converts the incident photons into an electrical signal, and a high-speed photon technology circuit accurately counts the photons. The data is finally transmitted to the computer through a USB 2.0 interface, where it is processed using the H11890 Series Sample Software Version 2.20 to generate time-resolved photon-counting curves, sampling the photon count at 1000 ms intervals to track absorbance over time.

## 3. Results

### 3.1. Optimization of Light Source Voltage and Calibration of Optical Detection Distance

**Figure 4:**
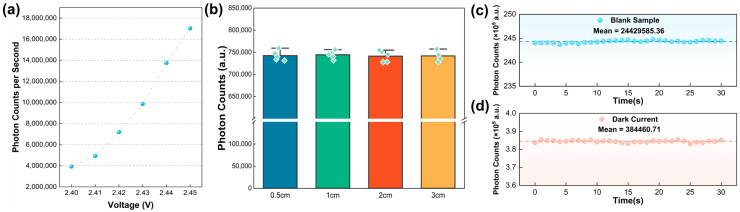
Optimization of detection method conditions and quantitative performance based on changes in absorbance. (**a**) The influence of different power supply voltages on the variation in photon counts per minute. (**b**) The relationship between the vertical distance between the detection module lens and the microfluidic chip and the changes in photon count per minute. (**c**) Measurement of the device’s dark current showed an average photon count of 384,460.71 ± 559.22. (**d**) In the absence of loaded droplets, the system’s average photon count was found to be 24,429,585.36 nm, with a coefficient of variation of 0.103%.

The voltage of a regulated DC power supply directly influences the stability of its light source output, which in turn determines the accuracy of absorbance detection. To establish the optimal operating voltage, this study utilized a 100% active AT standard as the test subject. Preliminary experiments indicated a significant decrease in PMT signal intensity when the voltage fell below 2.40 V. Consequently, the formal experimental voltage range was established as between 2.40 V and 2.45 V. A precise voltage stepping method was employed to systematically examine six characteristic voltage points: 2.40 V, 2.41 V, 2.42 V, 2.43 V, 2.44 V, and 2.45 V. The results are illustrated in Figure 4a. The experiments revealed that when the voltage exceeded 2.45 V, the PMT photon count reached saturation, causing feedback errors from the software. Conversely, when the voltage was ≤2.45 V, the baseline absorbance increased with rising voltage. Based on the principle of maximizing the signal-to-noise ratio, 2.44 V was ultimately selected as the optimal operating voltage. The vertical distance (Z-axis height) between the detection module lens and the microfluidic chip affects the light path’s focusing efficiency and signal acquisition intensity. To evaluate detection performance for the 100% active AT sample at varying heights within a range of 0.5 to 3.0 cm, experiments were conducted, and the results are displayed in Figure 4b. It was observed that the absolute absorbance exhibited exponential decay with increasing distance, while ΔA/min showed no significant differences across the different heights. To balance signal strength and equipment compactness, 1.0 cm was selected as the standard working distance. An assessment of background noise was performed, as shown in Figure 4c. Under completely dark conditions, the PMT continuously collected dark current signals over 30 time points at 1000 ms intervals, yielding an average photon count of 384,460.71 ± 559.22 (mean ± SD). This background noise was subsequently eliminated from the raw data using a real-time subtraction algorithm. As indicated in Figure 4d, during tests with an empty chip, where no droplets were loaded, the system’s average photon count was found to be 24,429,585.36, with a fluctuation range of less than ±0.5% (coefficient of variation, CV = 0.103%). These results demonstrate that the light source within the system exhibits exceptional stability, maintaining consistent output with negligible variability. To investigate the effect of temperature on the PMT module, we first referred to the PMT H11890-01 specifications, which indicated an operating temperature range of 5 °C to 40 °C. We then examined the feasibility of the PMT module within a selected range of 18 °C to 38 °C. By adjusting the ambient temperature using an air conditioner (without adding samples), we established the relationship between the temperature and detected photon count as y = 7704.504x + 2.358E7, as shown in Figure S4. The experimental results demonstrated that within the tested temperature range (18 °C, 23 °C, 28 °C, 33 °C, and 38 °C), temperature had a negligible influence on the absorbance detection module. Additionally, the PMT module’s dark current remained within 10^2^~10^3^, which had an insignificant impact on the measurement process.

### 3.2. Verification of Detection Accuracy and Method Feasibility

**Figure 5:**
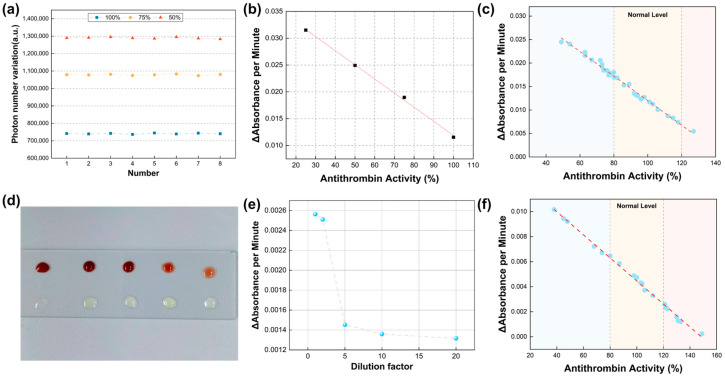
Qualitative detection of chip performance with clinical samples. (**a**) Variability measurements of photon count from plasma at three different activity levels (100%, 75%, and 50%) across eight replicate experiments, resulting in coefficients of variation of 3.64%, 2.97%, and 3.21%, respectively. (**b**) Reconstitution of lyophilized calibration standards, validating the feasibility of four concentrations: 100%, 75%, 50%, and 25%. (**c**) Relationship between activity levels and absorbance changes in 30 clinical samples (using plasma as the test sample), with the central yellow region indicating normal activity levels. (**d**) Visualization of blood and reagents at varying dilution concentrations; from left to right, the dilution factors are 1, 2, 5, 10, and 20. (**e**) Results of absorbance changes with different dilution factors. (**f**) Relationship between activity levels and absorbance changes in 20 clinical samples (using diluted venous whole blood as the test sample), with the central yellow region representing normal activity levels.

To demonstrate the reproducibility of the method, we prepared three batches of plasma with varying activity levels (100%, 75%, and 50%). The same operator performed eight repeated measurements on the same device, calculating the mean (M) and standard deviation (SD) of the results. The coefficient of variation (CV) was then determined using the formula CV (%) = (SD/M) × 100%. As shown in Figure 5a, the experimental results yielded coefficients of variation of 3.64%, 2.97%, and 3.21%, respectively. According to the description of the antithrombin III assay kit in the Chinese Pharmacopoeia [39], the repeatability of its assay results should meet the requirement of a coefficient of variation (CV) ≤10%. The above results meet this requirement, demonstrating a high level of result stability and good reproducibility for detecting anticoagulant activity in plasma with different activity levels. To further validate the feasibility of the proposed method for plasma sample analysis, we reconstituted freeze-dried calibrated anticoagulant samples (100% activity) using a designated buffer solution. We tested four concentration gradients: 100%, 75%, 50%, and 25%. By monitoring the dynamic changes in photon count during the thrombin reaction, as illustrated in Figure 5b, the data revealed significant kinetic differences among samples with varying activity levels. Specifically, higher activity levels were associated with slower rates of color reaction and a correspondingly reduced rate of photon count decline. These findings suggest that the dynamic variations in photon counts can serve as effective indicators of sample activity levels, further validating the feasibility of the proposed method for plasma sample detection.

### 3.3. Linear Relationships and Clinical Consistency

To further validate the clinical efficacy of this method, we tested plasma samples from 30 patients, comprising 14 samples within the normal activity range and 16 samples exhibiting abnormal activity. The test results, illustrated in Figure 5c, reveal a pronounced linear decline in the rate of change of absorbance with increasing antithrombin activity. The curve equation is expressed as y = 0.0382 − 0.02616x, with an R^2^ value of 0.990 (≥0.985), and the Pearson correlation coefficient is −0.995. These findings indicate a significant linear relationship in the spectral data, demonstrating a negative linear correlation between the level of antithrombin activity and the rate of change in absorbance over time. This result further confirms the effectiveness of this method in plasma sample detection, laying the foundation for subsequent venous whole blood and capillary blood measurements. Additionally, we conducted a Kolmogorov–Smirnov (KS) test on the results for clinical samples and calibrators, yielding a *p*-value of 0.9753. This suggests that the fitted curves for the two datasets are closely aligned, with no significant differences in their distributions, indicating commendable consistency in the results.

### 3.4. Validation of the Capability of Micro-Volume Venous Whole Blood Analysis

The use of blood plasma samples is time-consuming as they involve steps such as transport and centrifugation, which fail to meet the requirements of POCT testing. We attempted detection using venous whole blood. However, venous whole blood samples contain significant amounts of cellular components, particularly red blood cells, which interfere with the release of pNA due to their high hemoglobin content, thereby preventing the accurate detection of AT activity levels. To mitigate this interference, we employed a dilution approach. The observed effect is presented in Figure 5d. To identify an appropriate dilution factor, we tested dilutions of 1, 2, 5, 10, and 20 times. The experimental data, shown in Figure 5e, indicate that as the dilution factor increases, the change in absorbance decreases rapidly, and the magnitude of change stabilizes with greater dilution. Therefore, balancing the reagent volume and detection efficacy, we selected a dilution factor of 10. To achieve automation, we added whole blood droplets and factor diluent to the chip at a volume ratio of 1:9, followed by mixing according to our pre-programmed procedure. This process is illustrated in Figure S3. Following the outlined experimental procedure, we measured the changes in absorbance per minute and the corresponding AT activity levels. This method was applied to twenty patient samples, as illustrated in Figure 5f. The scatter results were subjected to linear regression analysis, yielding the fitted curve equation y = 0.01363 − 0.00916x, R^2^ = 0.995 ≥ 0.9850, and a Pearson correlation coefficient of −0.998. The resulting curve demonstrates a strong linear relationship, indicating that the measurement of AT activity levels is negatively correlated with the change in absorbance over time in micro-volume venous whole blood samples. The preliminary results suggest that the developed system shows promise for the detection of AT in whole venous blood samples.

### 3.5. Further Validation of AT Detection in Fingertip Capillary Blood by the Microfluidic System

To meet the requirements for POCT, such as home testing, venous whole blood is often insufficient because it needs to be collected by professionals and requires special blood collection tubes, which will greatly reduce efficiency. Fingertip capillary blood is often used in home monitoring because it is readily available and easy to handle. In the aforementioned experiments, venous whole blood yielded satisfactory detection results. Given that fingertip capillary blood shares largely identical components with venous blood, we hypothesize that it can also be utilized for detection. Therefore, we randomly selected 10 volunteers and simultaneously collected fingertip capillary blood and venous blood to further validate the stability and accuracy of the microfluidic system we developed for AT detection. The venous blood was processed following the aforementioned protocol, centrifuged, and then analyzed using a standard clinical instrument. The fingertip capillary blood was immediately tested using the microfluidic system after collection. The process for the automatic dilution of capillary blood is consistent with that used for whole blood in the vein. The results are presented in Table 1, demonstrating a high degree of consistency between the AT activity levels measured by our microfluidic system using capillary blood and those obtained with conventional large-scale laboratory instruments. The results demonstrate that the detection of AT can be achieved using fingertip capillary blood with our developed microfluidic system, which will significantly advance POCT for AT.

### 3.6. Further Validation of AT Detection in Special Specimens by the Microfluidic System

To evaluate the versatility of our microfluidic system for different blood sample types, we tested three special specimen categories: icteric, hemolyzed, and lipemic samples. The appearance of these samples is shown in Figure S2, and the test results are summarized in Table S1. Initially, we quantified total bilirubin, hemoglobin, and triglyceride levels in three distinct specimen types following the reagent manufacturer’s protocols. At the specified concentrations, the test results obtained using conventional laboratory instruments showed no interference. Subsequently, these measurements were replicated using the microfluidic system. The system we developed obtained better results in dealing with these challenging samples, which were similar to the data measured through a clinical routine laboratory procedure; however, the results for chyle samples were slightly poorer. We hypothesize that the interference of triglycerides with colorimetric changes may intensify when using smaller reagent volumes for measurement. In the future, we will optimize the detection system to reduce its potential impact on the accuracy of the test.

## 4. Discussion

AT plays a crucial role in the body’s anticoagulation process. The level of AT activity is not only correlated with the effectiveness of clinical anticoagulation treatments using heparin but also closely related to the screening of thromboembolic disorders and the diagnosis of liver and kidney diseases. However, conventional detection methods fail to meet the demands for high-throughput and rapid testing scenarios. Thus, there is an urgent need to develop a more efficient and convenient detection method to enhance clinical diagnosis and patient management. In this study, we developed a POCT system for AT detection based on digital microfluidics technology. This system does not rely on large, high-end equipment or specialized personnel; it offers ease of use and greater adaptability to various scenarios. The experimental data align closely with the results from conventional testing methods, confirming the feasibility and accuracy of our approach. Additionally, our research innovatively measures AT activity in micro-volume-capillary whole blood samples, employing a dilution method to minimize the interference of background coloration due to red blood cells on the experimental results. The experimental data demonstrate a high degree of linear consistency with conventional testing outcomes. The results reveal significant developmental prospects, as this method significantly reduces the required sample volume, allowing detection from micro-capillary blood. This advancement eliminates the need for professional blood draws, making it more suitable for home monitoring and bedside testing—a considerable benefit for patients who face difficulties with blood sampling.

Furthermore, the digital microfluidic system is characterized by its simplicity and high level of automation, moving away from dependence on large equipment and facilitating portability. This makes it particularly suitable for emergency field testing and primary care settings, thereby expanding its range of applications. We estimated the time required for our developed microfluidic system to process trace whole blood samples and obtain AT test results. The entire process takes approximately six minutes, with the detailed time distribution shown in Table S2. In contrast, conventional laboratory AT testing, including blood collection, sample transportation, and machine analysis, typically takes about 2–3 h. The system addresses several issues related to sample collection, reduces the costs of sample transportation management, and simplifies complex procedures such as whole blood centrifugation, consequently shortening the waiting time for results. Current POCT methods represent a focal point in the advancement of in vitro diagnostics. The AT activity detection method we have developed, leveraging digital microfluidics technology, aligns closely with the evolution of POCT. The use of micro-volume-capillary blood samples ensures convenience and ease of access, saving a great deal of time and enhancing the adaptability and operability of this detection method. Numerous advantages facilitate efficient and convenient clinical testing, highlighting significant potential for widespread applications.

The limitations of this study include the relatively small clinical specimen size, necessitating the expansion of the sample size in future investigations to further validate the developed microfluidic detection system. Furthermore, specific sample types, particularly chylous specimens, exhibit a pronounced tendency to interfere with chromogenic reactions and compromise assay accuracy. This also applies to the microfluidic system assay method we developed, and further optimization is still needed in the future to improve detection accuracy. The development of our system offers a novel approach to in vitro diagnostics. As technology advances, the further refinement of digital microfluidic platforms will enhance the application of this method in detecting other biomarkers, ultimately providing more efficient and convenient solutions for clinical diagnosis, thereby improving the accuracy and usability of clinical assessments.

## Figures and Tables

**Table 1:** Temporal changes in antithrombin activity and absorbance in fingertip capillary blood.

Sample ID	Regular Instrument AT Activity	Microfluidic System AT Activity	Calculated Relative Difference
1	113%	111.572%	1.2%
2	113%	113.100%	0.1%
3	112%	112.227%	0.2%
4	107%	109.716%	2.5%
5	104%	101.965%	1.9%
6	100%	96.834%	3.2%
7	100%	97.380%	2.7%
8	98%	95.306%	2.8%
9	94%	93.996%	0.0%
10	91%	92.467%	1.6%

## Data Availability

The datasets generated and/or analyzed during the current study are available from the corresponding authors upon reasonable request.

## Supplementary Materials

The following supporting information can be downloaded at https://www.mdpi.com/article/10.3390/mi16070785/s1: Figure S1. Digital microfluidic platform for antithrombin activity assay; Figure S2. Real photos of three special plasma specimens: (a) Bilirubin, (b) Hemolytic, (c) Chylous; Figure S3. Process diagram of whole blood automatic dilution on a microfluidic chip; Figure S4. Under the condition of no droplet addition, the absorbance module detection value under different ambient temperatures (temperature is 18℃, 23℃, 28℃, 33℃, 38℃). Table S1. The influence of hemolysis, lipidemia and jaundice on AT detection; Table S2. Timeline of the AT assay using digital microfluidics.
