# Supplementary material for: Automated Antithrombin Activity Detection with Whole Capillary Blood Based on Digital Microfluidic Platform
Source: Micromachines (Basel). 2025 Jun 30;16(7):785. doi: 10.3390/mi16070785 (PMC12298285; doi:10.3390/mi16070785)
Supplement: Supplementary file 1 [file micromachines-16-00785-s001.zip › micromachines-3678141-supplementary.pdf]

Supporting Information

Automated Antithrombin Activity Detection with Whole Capillary Blood Based on Digital Microfluidic Platform

Dongshuo Li [1,†], Hanqi Hu [2,†], Hanzhi Zhang [2], Lei Shang [1], Tao Zhao [2], Qingchen Zhao [2], Shuhao Zhang [2], Fucun Ma [1], Guowei Liang [1,*], Rongxin Fu [2,*] and Xuekai Liu [1,*]

[1] Department of Clinical Laboratory, Aerospace Center Hospital, Beijing 100049, China; lidongshuo1015@163.com (D.L.); leishamgsmile@foxmail.com (L.S.); mafucun721@126.com (F.M.)
[2] School of Medical Technology, Beijing Institute of Technology, Beijing 100081, China; 15258319540@163.com (H.H.); zhanghz20020608@163.com (H.Z.); 3220242885@bit.edu.cn (T.Z.); 13231821195@163.com (Q.Z.); zhangruanshi@163.com (S.Z.)
* Correspondence: lgw721@126.com (G.L.); furongxin@bit.edu.cn (R.F.); 15101129780@163.com (X.L.)
† These authors contributed equally to this work.

1. Experimental Setup

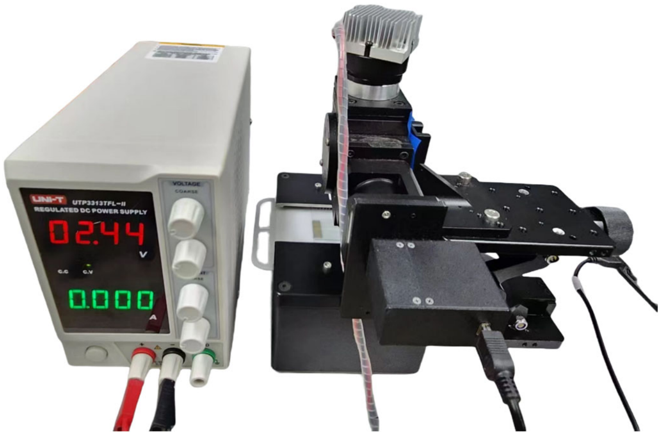

Figure S1. Digital microfluidic platform for antithrombin activity assay

2. The influence of special specimen types on AT determination

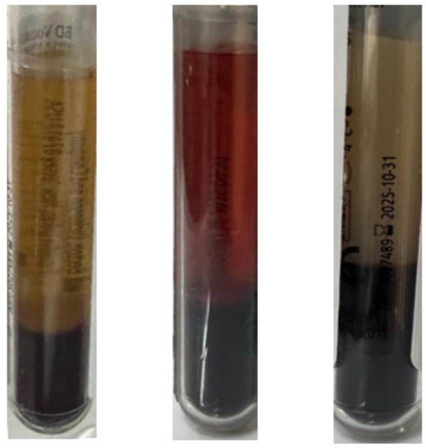

a b c

Figure S2. Real photos of three special plasma specimens: (a) Bilirubin, (b) Hemolytic, (c) Chylous

The antithrombin assay kit (chromogenic substrate method) REV202012 indicates that the results of antithrombin measured by ACL TOP are not affected by bilirubin up to 40

mg/dL, hemoglobin up to 500 mg/dL, and triglyides up to 2300 mg/dL. Firstly, we measured total bilirubin, hemoglobin and triglycerides in three special types of specimens, combined with the instructions for determination reagents, the results of routine laboratory instrument measurement at the existing concentration were not interfered, and then they were measured by microfluidic system. The system we developed has obtained better results in dealing with these challenging samples, which are close to the data measured by clinical routine laboratory, however, the results chyle samples are slightly poor.

Table S1. The influence of hemolysis, lipidemia and jaundice on AT detection

Sample	Lab-Measured AT Activity(%)	DMF-Measured AT activity(%)	Calculated Relative Difference	Total bilirubin content(mg/dL)	Hemoglobin content(mg/dL)	Triglyceride content(mg/dL)
A	89	91.132	2.4%	11.39＜40		
B	83	88.876	7.1%		200＜500	
C	115	128.708	11.9%			1063＜2300

3. Automated whole blood dilution process

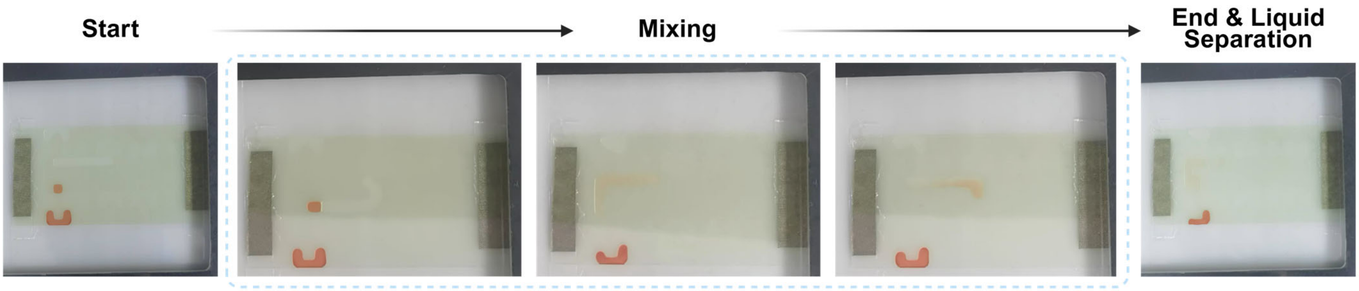

Figure S3. Process diagram of whole blood automatic dilution on a microfluidic chip.

4. Statistical analysis of AT determination time based on microfluidic system

Table S2. Timeline of the AT Assay Using Digital Microfluidics

Process	Step	Time
Specimen collection	Whole capillary blood collection	20s
Sample pretreatment	Automated dilution of whole capillary blood	120s
	The mixture of diluted whole capillary blood and factor diluent	20s
On-chip chromogenic reactions	Interaction with Factor Xa	100 s
	Chromogenic reaction	20 s

Absorbance detection	80 s
Total	360 s

5. Explore the feasibility of PMT modules under different temperature conditions

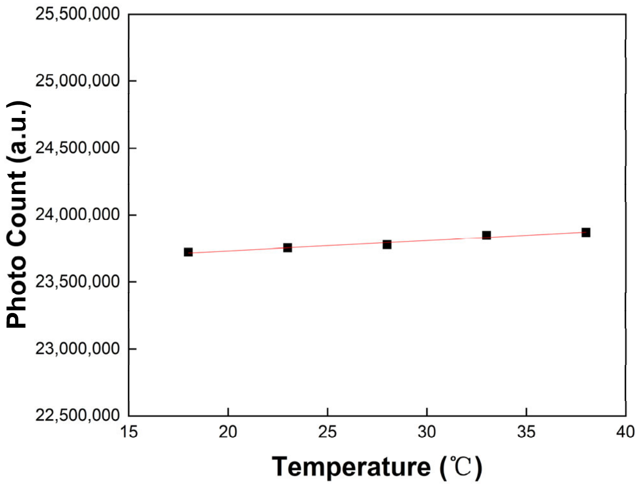

Figure S4. Under the condition of no droplet addition, the absorbance module detection value under different ambient temperatures (temperature is 18°C, 23°C, 28°C, 33°C, 38°C)
